# In-vitro evaluation of a novel MR-compatible cardiac bioptome catheter for MR-guided myocardial biopsies

**DOI:** 10.1186/1532-429X-14-S1-O32

**Published:** 2012-02-01

**Authors:** Sebastian A Seitz, Sebastian M Haberkorn, Herbert Maslanka, Hugo A Katus, Henning Steen

**Affiliations:** 1 Department of Cardiology, University of Heidelberg, Heidelberg, Germany; 2 H. + H. Maslanka Chirurgische Instrumente GmbH, Tuttlingen, Germany

## Background

Retrieval of myocardial biopsies under X-ray guidance in patients with unclear myocardial dysfunction or acute myocarditis is a difficult and potentially hazardous procedure since soft tissues only offer low X-ray contrast during the procedure and extraction of biopsies could cause myocardial rupture and concomitant haemopericardium. In contrast, cardiac magnetic resonance imaging provides excellent soft-tissue and anatomic information, especially for inflamed and fibrotic areas. MR-guided myocardial biopsies would therefore be attractive but could not be performed until now due to incompatible bioptome catheters leading to substantial image artifacts and significant device heating.

Evaluation a novel MR-compatible bioptome in in-vitro experiments to assess potential artifacts, safety aspects and performance of MR-guided navigation under real-time imaging in a cylindrical 1.5T system.

## Methods

**Figure 1:**
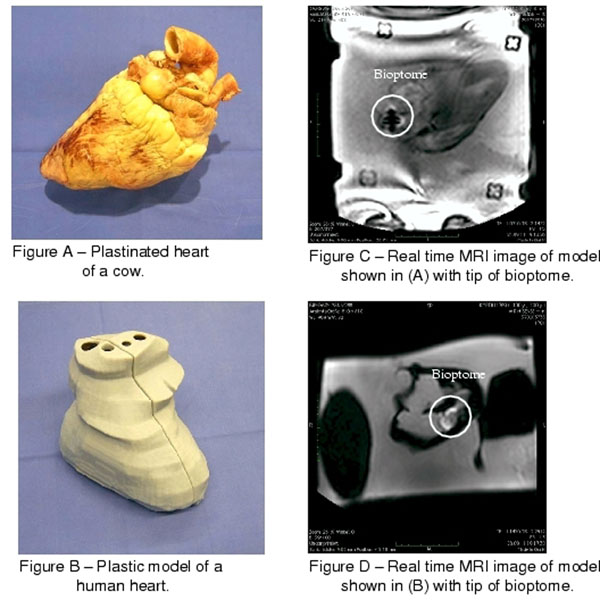


The bioptome was evaluated in a series of in-vitro experiments in an 1.5T MRI system (Philips Achieva). The specific device design (non-disclosure) avoided inducible currents and subsequent heating. The bioptome was introduced and navigated inside a) plastinated cow and swine hearts and b) a plastic heart model. The first model forms a true anatomic environment containing all relevant morphological structures (figure 1(A). The second model was derived from a human cardiac CT scan using rapid prototyping (figure 1(B)). Both phantom types were placed inside a saline filled plastic box. The MR-guidance was conducted with a continuous SSFP imaging sequence (frame rate=2 images/s) by visually tracking the artifact introduced by the metal element at the tip.

## Results

The metal tip of the bioptome produced an artifact that allowed constant and precise ex-vivo tip localization. The artifact size was approximately 3.3x3 cm (figure 1(C),1(D)). The different structural elements of the heart and the target regions for the biopsy were clearly visible enabling a significantly better navigation of the tip than in conventional X-ray. The shaft of the bioptome caused no artifacts.

## Conclusions

With the novel MR compatible bioptome, the superior CMR soft tissue visualization can be made available for MR-guided myocardial biopsies overcoming the limited soft tissue contrast on X-ray images. Clinically, this could significantly reduce the high amount of necessary specimen to overcome the sample error under X-ray and secondly improve the specificity and reliability of cardiac biopsies. Thirdly, MR-guided biopsies would minimize X-ray dose for the patient and especially the interventionalist.

## Funding

None.

